# Influence of Self-Adhesive Resin Composite Deep Marginal Elevation on the Sealing Ability of CAD/CAM Lithium Disilicate Glass-Ceramic Inlays: An In Vitro Study

**DOI:** 10.3390/polym18121555

**Published:** 2026-06-22

**Authors:** Rasha Haridy, Shadan Almotairi, Shoroug Alshehri, Abrar Nasser Bin Nooh, Moataz Elgezawi

**Affiliations:** 1 Department of Clinical Dental Sciences, College of Dentistry, Princess Nourah bint Abdulrahman University, P.O. Box 84428, Riyadh 11671, Saudi Arabia; 2 Department of Conservative Dentistry, Faculty of Dentistry, Cairo University, Cairo 4240310, Egypt; 3 Internship Program, College of Dentistry, Princess Nourah bint Abdulrahman University, Riyadh 11671, Saudi Arabia; shadanalmotairi@gmail.com (S.A.); shrog_98_19@hotmail.com (S.A.); 4 Dental Clinics Department, King Abdullah bin Abdulaziz University Hospital, Princess Nourah bint Abdulrahman University, Riyadh 11671, Saudi Arabia; analnooh@pnu.edu.sa; 5 Department of Restorative Dental Sciences, College of Dentistry, Imam Abdulrahman Bin Faisal University, P.O. Box 1982, Dammam 31441, Saudi Arabia; malgizawi@iau.edu.sa

**Keywords:** deep margin elevation, gap, self-adhesive resin composite, glass–ceramic inlay, lithium disilicate, micro-CT, CAD/CAM, cervical margin relocation

## Abstract

Deep margin elevation (DME) is a conservative technique used to relocate subgingival proximal margins to a more favorable supragingival position, facilitating adhesive procedures and impression taking. This in vitro study evaluated the influence of two DME materials—a universal flowable resin composite and a self-adhesive flowable resin composite—on the cervical interfacial sealing ability of lithium disilicate glass–ceramic CAD/CAM inlay restorations. Twenty extracted maxillary premolars were randomly allocated into two groups (*n* = 10). Group A received DME using a universal flowable resin composite (3M™ Filtek™ Z350 XT) preceded by a conventional adhesive system, while Group B received DME using a self-adhesive flowable resin composite (Vertise™ Flow). All teeth were restored with lithium disilicate CAD/CAM inlays (CEREC Tessera) cemented with a self-adhesive resin cement (Breeze^®^). Specimens underwent thermocycling (10,000 cycles; 5–55 °C). Marginal gaps were assessed at the DME interface using high-resolution micro-computed tomography (micro-CT) in both coronal and sagittal cross-sections, before and after thermocycling. Statistically significant differences were found between groups in both sections before and after thermocycling (*p* < 0.05). The self-adhesive composite (Group B) demonstrated significantly lower gap values compared to the universal flowable composite (Group A) in both coronal and sagittal assessments. Thermocycling increased the gap in both groups; however, Group B maintained considerably lower leakage. The self-adhesive resin composite showed superior sealing ability at the DME interface compared to the universal flowable composite when used under lithium disilicate glass–ceramic inlay restorations. Further clinical studies are recommended to validate these findings.

## 1. Introduction

Restorative treatment of deep sub-gingivally extending proximal caries poses a significant clinical challenge owing to the difficulty of managing deeply located margins. At such locations, enamel is absent, precluding the more favorable bonding conditions its presence would afford. Additionally, excessive moisture contamination remains a persistent risk, as field isolation and control of gingival tissue fluids beyond the cementoenamel junction (CEJ) are inherently difficult [1,2]. Deep margin elevation (DME) is a conservative alternative to surgical crown lengthening and orthodontic extrusion that has shown promise in managing deep proximal carious lesions [3]. The procedure provides a stepwise supragingival relocation of a subgingival proximal margin, facilitating both direct and indirect adhesive restorations [4].

The technique was first described by Taylor and Burns (2024), and it allowed coronal relocation of deep subgingival margins using a resin composite restoration to improve bonding quality for direct and indirect restorations [4]. DME offers several clinical benefits, including histological and periodontal health compatibility, preservation of the biological width, facilitation of impression taking, and enabling proper field isolation and optimized bonding procedures [5]. Alternative terminology for the same technique includes cervical margin relocation (CMR), the margin elevation technique (MET), and proximal box elevation [6]. The popularity of DME has grown significantly among general practitioners and specialists over the past decade [4]. A recent literature review by Samartzi et al. (2022) comprehensively summarized the current evidence on DME indications, techniques, and clinical outcomes, highlighting the critical importance of material selection and adhesive protocols for achieving marginal seal success [7]. Despite its growing adoption, a survey revealed that 78% of dentists remain uncertain about the clinical use of DME, with concerns regarding isolation efficacy, preservation of the biological width, marginal sealing, and the risk of microleakage. Limited high-quality clinical evidence continues to impede widespread confidence in the technique [4].

Self-adhesive flowable composites were introduced as a novel category combining properties of composites and self-etching adhesives, eliminating the need for a separate bonding agent application. Classified as the eighth generation of dentin adhesive systems, these materials offer advantages including simplified placement protocols, reduced postoperative sensitivity, better handling characteristics, and acceptable esthetics [8]. Understanding the sealing ability of materials used for DME is therefore of critical clinical relevance.

Computer-aided design and computer-aided manufacturing (CAD/CAM) technologies have achieved remarkable improvements in recent years, including advances in intraoral scanning precision, restorative material properties, and milling accuracy. Lithium disilicate reinforced glass–ceramic CAD/CAM inlays, cemented with self-adhesive resin luting cements, represent a clinically relevant restorative approach that has demonstrated superior fit, marginal precision, and long-term sealing potential [9,10]. Their recorded advantages include predictable marginal sealing, favorable esthetics, adequate strength, and biocompatibility in the oral environment [6]. Their favorable marginal adaptation and sealing ability have also been demonstrated in ceramic inlays luted with different resin cements [11].

Sealing against microleakage is essential for maintaining the biomechanical and esthetic integrity of any restoration. Microleakage can lead to postoperative hypersensitivity, secondary caries, adverse pulpal consequences, marginal discoloration, and ultimate restoration failure [12]. Micro-CT imaging has been validated as a reliable, non-destructive three-dimensional method for assessing marginal sealing ability and interfacial gap formation [13]. Notably, Theisen et al. (2023) used micro-CT to assess the quality of CAD/CAM inlays placed on aged resin-based composite restorations serving as DME, demonstrating that the integrity of the DME composite interface is a key determinant of overall restoration quality [14].

To date, comprehensive evidence comparing the sealing performance of self-adhesive and universal flowable composites as DME materials beneath lithium disilicate inlay restorations remains limited. This in vitro study therefore aimed to evaluate and compare the cervical interfacial sealing ability of lithium disilicate glass–ceramic inlay restorations when either a universal flowable resin composite or a self-adhesive flowable resin composite was used for DME. The null hypothesis was that there is no significant difference between the two DME materials with regard to the cervical interfacial sealing ability of glass–ceramic inlay restorations.

## 2. Materials and Methods

### 2.1. Ethical Approval

This in vitro study was conducted at the College of Dentistry, Princess Nourah bint Abdulrahman University (PNU), Riyadh, Saudi Arabia, following institutional review board approval (IRB No.: HAP-01-R-059).

### 2.2. Sample Collection and Preparation

Twenty mature, caries-free human maxillary premolars extracted for orthodontic reasons were recruited for this study. Inclusion criteria comprised sound, average-sized teeth free of cracks and developmental defects, as verified under 3.5× magnification dental loupes. Teeth were disinfected with 5.25% sodium hypochlorite for 30 min and subsequently stored in a 0.1% thymol solution (Sigma-Aldrich, St. Louis, MO, USA) at room temperature until use. Specimens were selected to fall within defined dimensional ranges (average mesiodistal crown width: 7.0 ± 0.5 mm; buccolingual width: 9.0 ± 0.5 mm), as verified by the pre-preparation digital scans. It is acknowledged that patient demographic data (age, gender) and extraction-to-storage intervals were not formally recorded for individual specimens; all teeth were stored under a consistent protocol, and their extraction for orthodontic reasons implies a relatively homogeneous donor population. Future studies should document and report specimen demographic data to enable more rigorous standardization.

### 2.3. Experimental Groups and Sample Size Calculation

Teeth were randomly divided into two groups of ten specimens each (*n* = 10). In Group A (control), a universal flowable resin composite (3M™ Filtek™ Z350 XT Flowable Restorative, 3M ESPE, St. Paul, MN, USA) was used for DME following conventional adhesive application. In Group B (experimental), a self-adhesive flowable resin composite (Vertise™ Flow, Kerr Corporation, Brea, CA, USA) was employed for DME without a separate bonding step. The sample size was calculated by a statistician based on a significance level of 0.05, a confidence level of 95%, and a statistical power of 80%. Materials used in this study are summarized in Table 1.

### 2.4. Pre-Preparation Baseline Scanning

All teeth were scanned using a Primescan intraoral scanner (Dentsply Sirona CEREC, Charlotte, NC, USA) prior to cavity preparation to record the original tooth anatomy. The baseline scan of each specimen was saved in the CEREC software (version 5.2.7), to be used as reference during inlay design following cavity preparation.

### 2.5. Cavity Preparation

All 20 specimens were mounted in quick-setting heat-curing pink acrylic resin (ECO-CRYL HOT, Protechno, Vilamalla, Spain) using a universal mounting device, at a level 3 mm apical to the CEJ. To minimize operator variability, cavity preparations were performed by a single investigator using a high-speed contra-angle handpiece (400,000 rpm; T3 Turbine, Sirona, Bensheim, Germany) with round carbide and round-end tapered diamond burs (FG:6 #25 mm, TR-13, ISO 198/018, Mani, Tokyo, Japan), replacing the bur for each tooth.

Standardized class II MOD cavities were prepared with the following dimensions: 2 mm occlusal depth, occlusally divergent buccal and lingual walls (5–10°), 3 mm buccolingual occlusal width, 4 mm buccolingual proximal width, rounded internal line angles, and butt-joint cavosurface margins. In one proximal box of each tooth, the gingival margin was positioned 1 mm coronal to the CEJ, while in the contralateral proximal box, it was positioned 2 mm apical to the CEJ to simulate a subgingival margin requiring DME.

### 2.6. Deep Marginal Elevation (DME) Procedure

A Kerr 2181 Adapt Super Cap matrix (steel, 0.038 mm thick, 5.0 mm high) was applied to each prepared tooth, ensuring the apical end of the matrix extended below the deep proximal gingival margin to facilitate DME. Matrix band application was performed consistently by a single operator throughout the study; however, the contact pressure of the matrix band was not formally measured or quantified, representing a methodological limitation. Future studies should consider using standardized tightening protocols or automated application devices to provide more rigorous control of this variable.

In Group A, selective etching of the gingival and axial walls at the DME site was performed using 37% phosphoric acid gel (Meta Etchant, Meta Biomed, Cheongju-si, Republic of Korea) for 30 s, followed by water rinsing for 30 s and gentle air drying. A universal adhesive (3M™ Adper™ Universal Single Bond, 3M ESPE, St. Paul, MN, USA) was applied with a microbrush, air-thinned for 3 s to evaporate the solvent, and light-cured for 20 s using an LED light-curing unit (Elipar™ DeepCure-S, 3M ESPE, St. Paul, MN, USA; 1200 mW/cm^2^). The universal flowable composite (3M™ Filtek™ Z350 XT) was then applied in two successive horizontal increments of approximately 2 mm each, light-cured for 20 s per increment, to relocate the cervical margin approximately 2 mm coronal to the CEJ. For all light-curing steps, the light-curing unit tip was positioned perpendicular to the restoration surface and maintained at approximately 1 mm, consistent across all specimens and performed by a single operator.

In Group B, the self-adhesive flowable composite (Vertise™ Flow, Kerr Corporation, USA) was applied directly to the conditioned cavity surfaces—without a separate adhesive step—in two successive increments, each light-cured for 20 s, following the same margin relocation protocol as Group A (Table 1).

### 2.7. Optical Impression and CAD/CAM Inlay Fabrication

Following DME, all teeth were digitally scanned using the Primescan scanner. Inlay designs were generated using CEREC software (Dentsply Sirona CEREC, NC, USA) referencing the pre-preparation baseline scans. High-strength advanced lithium disilicate CEREC Tessera CAD/CAM blocks (Dentsply Sirona, NC, USA) were used to mill the inlays using a CEREC Primemill unit (Dentsply Sirona, NC, USA). Milled inlays underwent a firing cycle in a CEREC SpeedFire furnace (Dentsply Sirona) to achieve full crystallization (850 °C; 3 min heat ramp, 1 min holding, 1 min cooling). The intaglio surfaces were treated with 10% hydrofluoric acid (Angelus, Londrina, Brazil) for 20 s to enhance micromechanical retention, followed by application of a silane coupling agent (RelyX™ Ceramic Primer, 3M ESPE, USA).

### 2.8. Cementation

Inlays were cemented using a dual-cure, self-adhesive resin cement (Breeze^®^, Pentron Clinical, USA; shade A2) applied under a single investigator’s finger load. Excess cement was removed with a plastic instrument, followed by light curing for 20 s per surface.

### 2.9. Thermocycling (Aging)

All specimens were subjected to 10,000 thermocycles using a thermocycling machine (SD Mechatronik, Westerham, Germany) to simulate clinical aging, with temperature alternating between 5 °C and 55 °C, a dwell time of 30 s per bath, and a transfer time of 5 s.

### 2.10. Gap Analysis by Micro-CT

Specimens were mounted in a micro-positioning stage and scanned using a Bruker SkyScan 1172 high-resolution micro-CT unit (Bruker SkyScan, Kontich, Belgium; hardware version A, software version 1.6) with the following acquisition parameters: 92 kV voltage, 108 µA anode current, 316 ms exposure time, 15 µm pixel size, Al + Cu filter, 0.5° rotation step over 360°, frame averaging of 4, and random movement of 8 to minimize ring artifacts. Total scanning time was approximately 46 min, with a matrix resolution of 524 × 1000. A flat-field correction was performed prior to scanning.

Reconstructed cross-sections were generated using NRecon software (version 1.6.9.4; Bruker SkyScan, Kontich, Belgium). Applied correction parameters included ring artifact reduction (value = 5), beam hardening compensation (25%), and Gaussian smoothing (kernel = 2). Images were saved as 16-bit TIFF files. Pre- and post-thermocycling reconstructed datasets were co-registered and analyzed using DataViewer software (version 1.5.6.2; Bruker SkyScan). Gap was quantified in millimeters at the DME interface in both coronal and sagittal cross-sections, before and after thermocycling.

### 2.11. Statistical Analysis

All gap measurements (mm) were tabulated and analyzed using appropriate statistical software. Paired t-tests were applied to compare gap values before and after thermocycling within each group. Independent-samples t-tests were used to compare gap between the two groups at each time point. A significance level of *p* ≤ 0.05 was set for all comparisons.

## 3. Results

The present study evaluated the sealing ability of two DME materials in conjunction with lithium disilicate glass–ceramic CAD/CAM inlay restorations. No gap was detected at the inlay–cement interface regardless of gingival margin location; therefore, all gap analyses were confined to the interface between the tooth structure (enamel or dentin) and the resin composite used for DME. All measurements are reported in millimeters (mm).

### 3.1. Gap Analysis in Group A (Universal Flowable Composite)—Coronal Section

**Figure 1:**
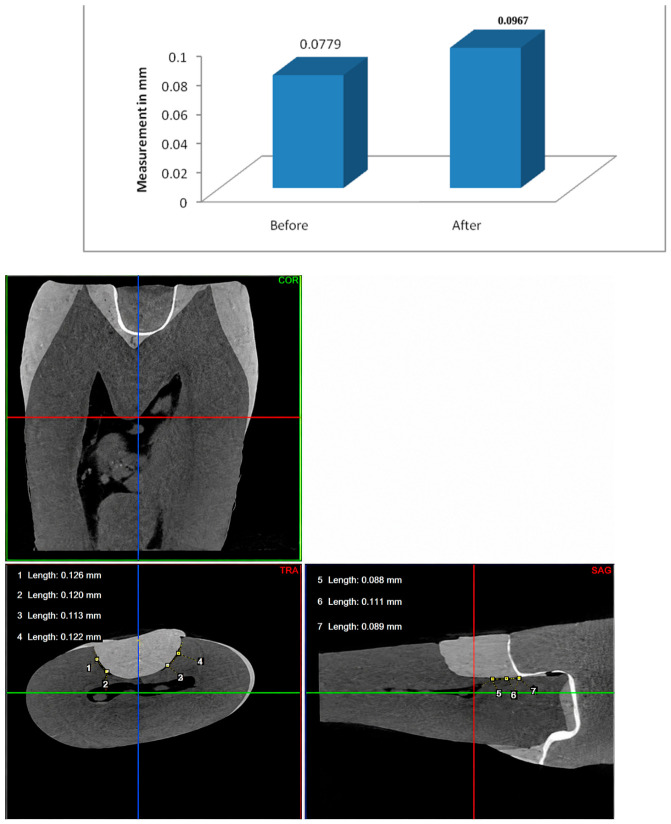
Group A—coronal cross-section. (**Upper panel**): bar chart showing mean marginal gap (mm) before and after thermocycling. (**Lower panels**): representative micro-CT images of the DME interface before thermocycling (**left**) and after thermocycling (**right**), with gap measurements indicated.

Table 2 and Figure 1 summarize the gap in Group A before and after thermocycling in the coronal section. The mean gap before thermocycling was 0.0779 ± 0.0276 mm, which increased to 0.0967 ± 0.0311 mm after thermocycling. This difference was statistically highly significant (*p* < 0.001).

### 3.2. Gap Analysis in Group A (Universal Flowable Composite)—Sagittal Section

**Figure 2:**
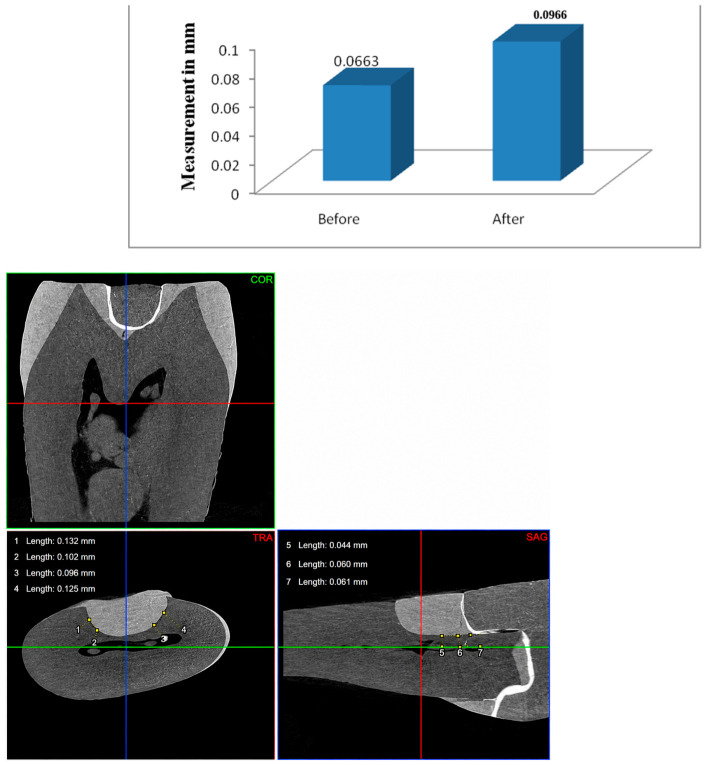
Group A—sagittal cross-section. (**Upper panel**): bar chart showing mean marginal gap (mm) before and after thermocycling. (**Lower panels**): representative micro-CT images at the DME interface before thermocycling (**left**) and after thermocycling (**right**), with gap measurements indicated.

Table 3 and Figure 2 present a gap in Group A in the sagittal section. The mean gap before thermocycling was 0.0663 ± 0.0453 mm and increased to 0.0966 ± 0.0294 mm after thermocycling. This increase was statistically highly significant (*p* < 0.001).

### 3.3. Gap Analysis in Group B (Self-Adhesive Composite)—Coronal Section

**Figure 3:**
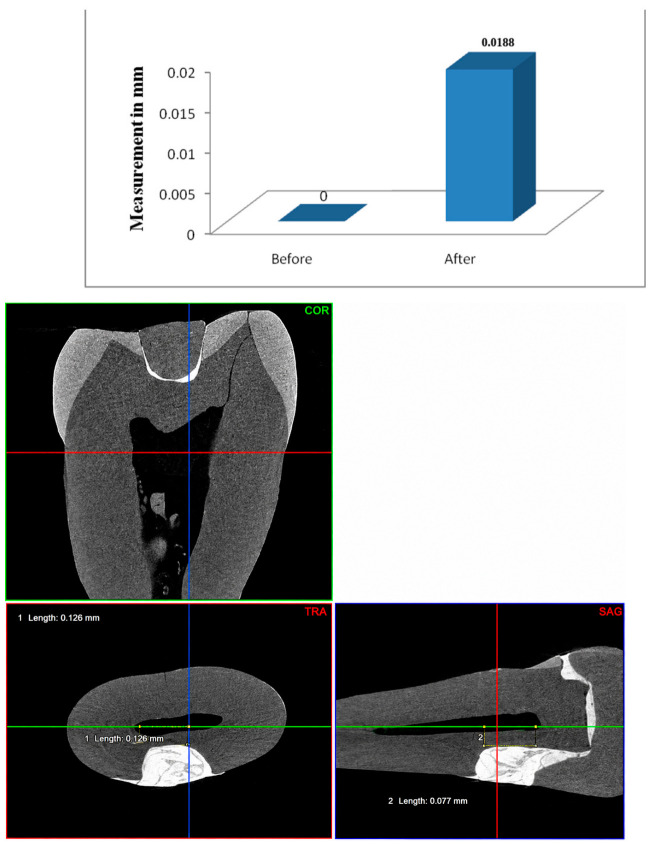
Group B—coronal cross-section. (**Upper panel**): bar chart showing mean marginal gap (mm) before and after thermocycling. (**Lower panels**): representative micro-CT images at the DME interface before thermocycling (**left**) and after thermocycling (**right**). The “NO GAP” label in the pre-thermocycling image confirms the complete absence of an interfacial gap.

Table 4 and Figure 3 show gap results for Group B in the coronal section. No gap was detected before thermocycling (0.0000 ± 0.0000 mm). After thermocycling, a mean gap of 0.0188 ± 0.0457 mm was recorded. Statistical comparison before and after thermocycling was not applicable due to the zero baseline variance.

### 3.4. Gap Analysis in Group B (Self-Adhesive Composite)—Sagittal Section

**Figure 4:**
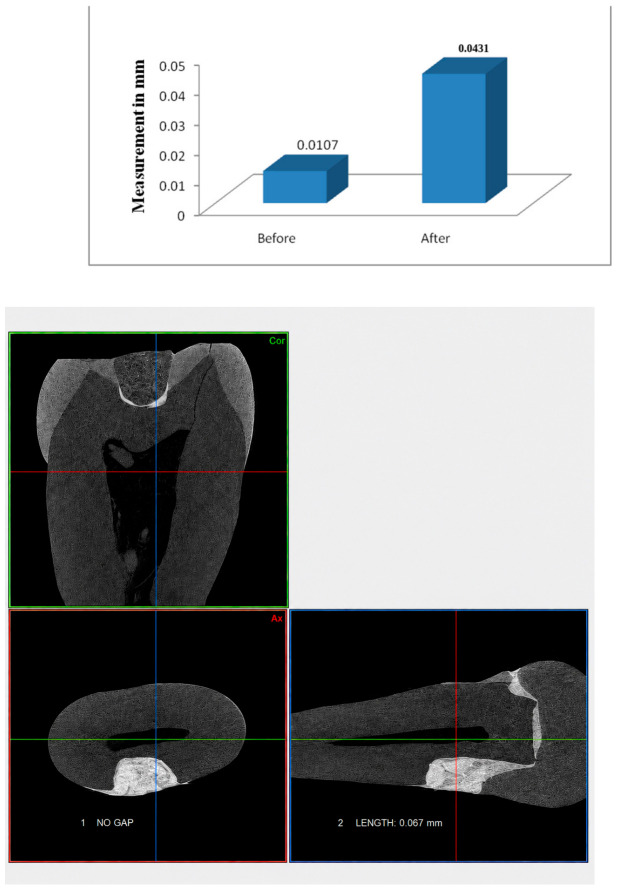
Group B—sagittal cross-section. (**Upper panel**): bar chart showing mean marginal gap (mm) before and after thermocycling. (**Lower panels**): representative micro-CT images of the DME interface before thermocycling (**left**) and after thermocycling (**right**). The “NO GAP” label in the pre-thermocycling image confirms the absence of an interfacial gap before aging.

Table 5 and Figure 4 present the gap in Group B in the sagittal section. Before thermocycling, the mean gap was 0.0107 ± 0.0267 mm, which increased significantly to 0.0431 ± 0.0492 mm after thermocycling (*p* = 0.001).

### 3.5. Comparison Between Groups—Coronal Section

**Figure 5:**
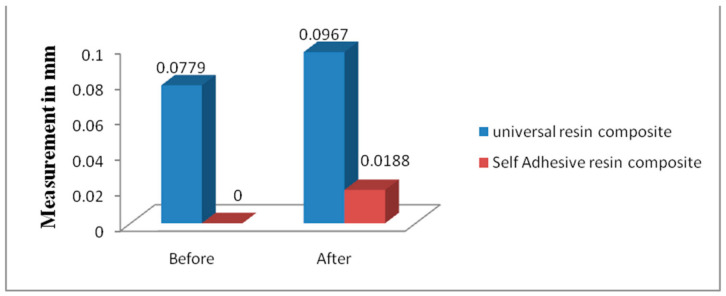
Intergroup comparison—coronal cross-section. Bar chart showing mean marginal gap (mm) for Group A (universal flowable resin composite) and Group B (self-adhesive flowable resin composite) before and after thermocycling.

Table 6 and Figure 5 compare the gap between groups in the coronal section. Before thermocycling, Group A recorded a mean gap of 0.0779 mm compared to 0.0000 mm in Group B (*p* < 0.001). After thermocycling, Group A (0.0967 mm) continued to exhibit significantly greater leakage than Group B (0.0188 mm; *p* < 0.001). In both time points, the self-adhesive composite group demonstrated statistically superior sealing compared to the universal composite group.

### 3.6. Comparison Between Groups—Sagittal Section

**Figure 6:**
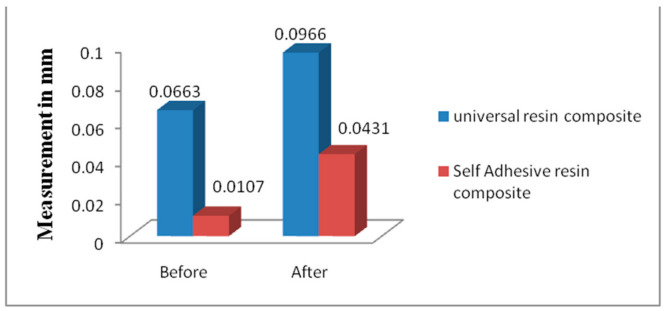
Intergroup comparison—sagittal cross-section. Bar chart showing mean marginal gap (mm) for Group A (universal flowable resin composite) and Group B (self-adhesive flowable resin composite) before and after thermocycling.

Table 7 and Figure 6 summarize the intergroup comparison in the sagittal section. Before thermocycling, Group A exhibited a significantly higher mean gap (0.0663 mm) compared to Group B (0.0107 mm; *p* < 0.001). After thermocycling, Group A (0.0966 mm) remained significantly higher than Group B (0.0431 mm; *p* < 0.001), confirming the superior marginal sealing of the self-adhesive composite in both planes of the section.

## 4. Discussion

Deep margin elevation is recognized as a viable conservative alternative to surgical procedures for managing teeth with subgingival margins. By converting the operational environment from subgingival to supragingival, DME addresses multiple clinical challenges simultaneously: isolation, impression taking, and bonding of indirect restorations. The technique was first proposed by Taylor and Burns (2024) and has since gained broad acceptance in clinical practice [3,4,15].

The primary concerns associated with DME relate to the quality of marginal adaptation and interfacial sealing. A persistent gap at the DME interface can permit ingress of oral fluids and microorganisms, leading to postoperative sensitivity, secondary caries, and restoration failure [12,16]. Consequently, the sealing ability of the DME material is critical to the overall success of the procedure. A Persistent gap at the DME interface may also contribute to recurrent and residual caries, further emphasizing the importance of durable marginal sealing [17].

Microleakage tests are among the most widely used in vitro methods to evaluate the performance of restorative materials, and while they carry inherent limitations, they are considered informative predictors of in vivo clinical performance [18,19]. In the present study, gap Analysis was quantified using high-resolution micro-CT, a validated three-dimensional, non-destructive approach that enables precise spatial analysis of interfacial gaps at the DME site [13]. This approach offers advantages over conventional dye-penetration methods used in some prior studies; for example, Reddy et al. (2024) evaluated microleakage and interface integrity in DME using confocal laser microscopy and scanning electron microscopy, methods that, while informative, are destructive and do not allow three-dimensional volumetric quantification of gap formation [20]. The results demonstrated that the self-adhesive flowable composite (Group B; Vertise™ Flow) exhibited significantly lower gap than the universal flowable composite (Group A; 3M™ Filtek™ Z350 XT) in both coronal and sagittal sections, both before and after thermocycling (*p* < 0.001). These findings reject the null hypothesis and are consistent with those of Juloski, Köken, and Ferrari (2020), who similarly reported superior sealing for self-adhesive composites compared to conventional flowable composites in analogous cavity configurations [21].

The superior performance of the self-adhesive composite may be attributed to its unique chemical composition. Vertise™ Flow contains glycerophosphate dimethacrylate (GPDM), a functional monomer that interacts directly with hydroxyapatite at the tooth surface, facilitating self-etching adhesion without the need for a separate bonding agent. This simplified adhesive interface may reduce technique-sensitive steps and minimize the risk of errors that can compromise marginal integrity [8,22].

Before thermocycling, no gap was detected at the self-adhesive composite interface in the coronal section, and only minimal leakage was observed in the sagittal section. In contrast, the universal composite group exhibited measurable gaps in both sections from the outset, despite the use of a separate adhesive system. This initial superiority of the self-adhesive material is consistent with the findings of Yuan et al. (2007) and supports the hypothesis that GPDM-based monomers provide effective early-stage marginal sealing [22].

Thermocycling significantly increases gap in both groups, as expected given the thermal stresses imposed on the adhesive interface and the associated dimensional changes in dental tissues and restorative materials. However, the self-adhesive composite maintained substantially lower gap values after aging compared to the universal composite in both measurement planes. This pattern is consistent with the stress-buffering capacity attributed to the elastic modulus characteristics of self-adhesive composites and their chemical bonding to tooth substrate [21,22].

A contrasting perspective is offered by Koeken et al. (2019), who found no statistically significant difference in gap between universal flowable and self-adhesive composites in a DME scenario, while noting that the enamel–adhesive interface consistently demonstrated superior sealing compared to dentin [23]. The discrepancy between their findings and those of the present study may reflect differences in specimen preparation, thermocycling protocols, and measurement methodology. Notably, the use of micro-CT in the present study allows volumetric, three-dimensional gap quantification rather than relying on linear dye penetration, which may account for the greater sensitivity of the current findings. More specifically, Koeken et al. (2019) [23] used microleakage dye penetration as the evaluation method, which is inherently qualitative and subject to observer bias, whereas the present study used high-resolution micro-CT with a spatial resolution of 15 µm, enabling precise, three-dimensional, quantitative gap measurement. This difference in measurement sensitivity may explain why the current study detected statistically significant intergroup differences that the dye penetration method failed to resolve. Additionally, differences in thermocycling cycles, restorative materials, cavity configurations, and luting cement systems between the two studies may have independently contributed to the divergent outcomes. Taken together, these discrepancies underscore the importance of evaluation methodology when comparing sealing studies and highlight the superior discriminative capacity of micro-CT analysis over conventional dye penetration. The positive influence of proximal box elevation on fracture resistance and microleakage of ceramic restorations has likewise been reported in premolars restored with ceramic endocrowns [24].

Regarding the use of self-adhesive resins in DME, it has been reported that these materials exhibit comparatively lower shear bond strength to dentin than conventional flowable composites with adhesive systems [8]. However, the present study suggests that adequate marginal sealing is achievable with self-adhesive composites at clinically relevant gap dimensions, despite this mechanical limitation. These results indicate that the functional performance in terms of sealing ability may not directly correlate with bond strength values measured under ideal laboratory conditions. The present study evaluated clinical performance with respect to gap formation and sealing ability; quantitative characterization of the adhesive efficacy of GPDM via bond strength testing or nanoleakage analysis was outside the scope of this investigation and represents an important avenue for future research.

It is important to acknowledge the inherent limitations of in vitro studies. The artificial aging through thermocycling, while standardized, may not fully replicate the complexity of the oral environment, including masticatory loading, salivary influences, and microbial pressure. Furthermore, the relatively small sample size (*n* = 10 per group) warrants cautious extrapolation to clinical settings. Future investigations should incorporate larger sample sizes, fatigue loading, and ideally clinical prospective trials to corroborate these laboratory findings. Additionally, the thermocycling protocol simulates only thermal stress and does not incorporate masticatory forces or fatigue loading, which are significant clinical stressors; future studies should integrate dynamic loading protocols. The current in vitro model did not simulate gingival fluid contamination or moisture exposure during DME application, a critical clinical variable; future studies should incorporate standardized moisture-contamination protocols to better reflect in vivo conditions. All cavity preparations and clinical procedures were performed by a single experienced investigator, minimizing intra-study variability but limiting generalizability to clinicians with varying skill levels; multi-operator studies would be valuable. The findings are specific to the tested CAD/CAM ceramic system (CEREC Tessera) and the subgingival margin depth of 2 mm apical to the CEJ; their applicability to other ceramic systems and deeper subgingival margins remains to be evaluated in future studies.

A clinically relevant consideration not addressed in the present study is the ease of DME material removal and reparability in cases of restoration failure. Self-adhesive composites, owing to their chemical interaction with dentin via phosphate-based monomers, may exhibit different debonding behavior compared to conventional flowable composites. Future clinical and laboratory studies should evaluate the removability, repairability, and retreatment implications of different DME materials, as these factors can meaningfully influence material selection in clinical practice. Furthermore, additional clinical and in vitro studies are recommended to explore additional performance parameters of these materials in DME, including shear bond strength, durability under masticatory loading, and long-term clinical reliability, and to validate these in vitro findings in prospective clinical trials [7,14,20].

## 5. Conclusions

Based on the findings of this in vitro study, the following conclusions are drawn:

1. The self-adhesive resin composite (Vertise™ Flow) demonstrated significantly superior cervical interfacial sealing ability compared to the universal flowable resin composite (3M™ Filtek™ Z350 XT) when used as a deep margin elevation material beneath lithium disilicate glass–ceramic CAD/CAM inlay restorations, in both coronal and sagittal cross-sections.
2. Thermocycling significantly increased the gap at the DME interface in both groups; however, the self-adhesive composite consistently maintained lower leakage values after artificial aging.
3. No gap was detected at the inlay–cement interface in either group, suggesting that the sealing performance of the self-adhesive resin cement was adequate regardless of the DME material used.
4. Further clinical and in vitro studies are recommended to explore additional performance parameters of these materials in DME, including shear bond strength, durability under masticatory loading, and long-term reliability, as well as to validate these findings in vivo.

## Figures and Tables

**Table 1:** Materials used in the study.

Material	Composition/Description	Manufacturer	Batch/Serial No.
3M™ Filtek™ Z350 XT Flowable Restorative	Resin Matrix: bis-GMA, UDMA, TEGDMA, bis-EMA, PEGDMA. Fillers: Silica nanoparticles (~20 nm), Zirconia nanoparticles (4–11 nm), Silica/Zirconia nanoclusters. Filler Loading: ~78.5 wt%/63.3 vol%	3M ESPE, St. Paul, MN, USA	N509855
Vertise™ Flow (Self-Adhesive Fluid Composite)	Resin Matrix: GPDM, HEMA, TEGDMA, Bis-GMA, UDMA. Fillers: 70 wt% (47 vol%) barium glass fillers, nanosized colloidal silica, ytterbium fluoride. Self-adhesive property due to GPDM monomer	Kerr Corporation, Brea, CA, USA	5842135
CEREC Tessera CAD/CAM Block	Advanced lithium disilicate block for chairside CAD/CAM restorations	Dentsply Sirona, Charlotte, NC, USA	16007898
3M ESPE RelyX™ Ceramic Primer	Silane coupling agent dissolved in organic solvent; used for conditioning ceramic surfaces before bonding	3M ESPE Dental Products, St. Paul, MN, USA	528374A
Breeze^®^ Self-Adhesive Resin Cement (Shade A2)	Methacrylate resin matrix (UDMA, TEGDMA, Bis-GMA derivatives); radiopaque fillers; adhesive monomers (phosphoric acid methacrylates)	Pentron Clinical, Orange, CA, USA	1904032

**Table 2:** Gap Analysis in Group A (universal flowable composite) before and after thermocycling—coronal section.

Group	Mean (mm)	Std. Deviation	Std. Error Mean	t Value	*p* Value
Before thermocycling	0.0779	0.02757	0.00453	7.540	<0.001 **
After thermocycling	0.0967	0.03106	0.00511		

** Highly significant (HS); *p* < 0.001.

**Table 3:** Gap Analysis in Group A (universal flowable composite) before and after thermocycling—sagittal section.

Group	Mean (mm)	Std. Deviation	Std. Error Mean	t Value	*p* Value
Before thermocycling	0.0663	0.04527	0.00716	8.376	<0.001 **
After thermocycling	0.0966	0.02941	0.00465		

** Highly significant (HS); *p* < 0.001.

**Table 4:** Gap Analysis in Group B (self-adhesive composite) before and after thermocycling—coronal section.

Group	Mean (mm)	Std. Deviation	Std. Error Mean	t Value	*p* Value
Before thermocycling	0.0000	0.00000	0.00000	—	—
After thermocycling	0.0188	0.04567	0.00807		

**Table 5:** Gap Analysis in Group B (self-adhesive composite) before and after thermocycling—sagittal section.

Group	Mean (mm)	Std. Deviation	Std. Error Mean	t Value	*p* Value
Before thermocycling	0.0107	0.02665	0.00513	3.831	0.001 *
After thermocycling	0.0431	0.04918	0.00947		

* Significant (S); *p* < 0.05.

**Table 6:** Comparison of Gap Analysis between Group A and Group B—coronal section.

	Group	Mean (mm)	Std. Deviation	Std. Error Mean	t Value	*p* Value
Before	Group A (universal)	0.0779	0.02757	0.00453	15.96	<0.001 **
	Group B (self-adhesive)	0.0000	0.00000	0.00000		
After	Group A (universal)	0.0967	0.03106	0.00511	8.368	<0.001 **
	Group B (self-adhesive)	0.0188	0.04567	0.00807		

** Highly significant (HS); *p* < 0.001.

**Table 7:** Comparison of Gap Analysis between Group A and Group B—sagittal section.

	Group	Mean (mm)	Std. Deviation	Std. Error Mean	t Value	*p* Value
Before	Group A (universal)	0.0663	0.04527	0.00716	5.736	<0.001 **
	Group B (self-adhesive)	0.0107	0.02665	0.00513		
After	Group A (universal)	0.0966	0.02941	0.00465	5.568	<0.001 **
	Group B (self-adhesive)	0.0431	0.04918	0.00947		

** Highly significant (HS); *p* < 0.001.

## Data Availability

The original contributions presented in this study are included in the article. Further inquiries can be directed to the corresponding author.

## Abbreviations

The following abbreviations are used in this manuscript:

CAD/CAM	Computer-aided design and computer-aided manufacturing
CEJ	Cementoenamel junction
CMR	Cervical margin relocation
DME	Deep margin elevation
GPDM	Glycerophosphate dimethacrylate
HEMA	Hydroxyethyl methacrylate
Micro-CT	Micro-computed tomography
MOD	Mesio-occluso-distal
MET	Margin elevation technique
